# A variety of hydrogenotrophic enrichment cultures catalyse cathodic reactions

**DOI:** 10.1038/s41598-018-38006-3

**Published:** 2019-02-20

**Authors:** Soroush Saheb-Alam, Frank Persson, Britt-Marie Wilén, Malte Hermansson, Oskar Modin

**Affiliations:** 1 Chalmers University of Technology, Department of Architecture and Civil Engineering, Division of Water Environment Technology, SE-412 96 Gothenburg, Sweden; 2 University of Gothenburg, Chemistry and Molecular Biology, SE-40530 Gothenburg, Sweden

## Abstract

Biocathodes where living microorganisms catalyse reduction of CO_2_ can potentially be used to produce valuable chemicals. Microorganisms harbouring hydrogenases may play a key role for biocathode performance since H_2_ generated on the electrode surface can act as an electron donor for CO_2_ reduction. In this study, the possibility of catalysing cathodic reactions by hydrogenotrophic methanogens, acetogens, sulfate-reducers, denitrifiers, and acetotrophic methanogens was investigated. The cultures were enriched from an activated sludge inoculum and performed the expected metabolic functions. All enrichments formed distinct microbial communities depending on their electron donor and electron acceptor. When the cultures were added to an electrochemical cell, linear sweep voltammograms showed a shift in current generation close to the hydrogen evolution potential (−1 V versus SHE) with higher cathodic current produced at a more positive potential. All enrichment cultures except the denitrifiers were also used to inoculate biocathodes of microbial electrolysis cells operated with H^+^ and bicarbonate as electron acceptors and this resulted in current densities between 0.1–1 A/m^2^. The microbial community composition of biocathodes inoculated with different enrichment cultures were as different from each other as they were different from their suspended culture inoculum. It was noteworthy that *Methanobacterium* sp. appeared on all the biocathodes suggesting that it is a key microorganism catalysing biocathode reactions.

## Introduction

Limitation of conventional energy sources and their impact on our climate, ecosystems and health has motivated a development of technologies that harvest renewable energy and allow environmentally friendly production of chemicals. The microbial electrolysis cell (MEC) is a modern, sustainable and promising approach to decrease electrode overpotentials, reduce the need for expensive metals catalysts, and allow direct generation of energy carriers at the cathode. MECs combine electrochemical systems with the catalytic ability of microorganisms for production of hydrogen^1–4^ and other valuable chemicals such as acetate^5,6^, methane^7–9^, caproate^10^, and alcohols^11^.

In MECs, microorganisms catalyse reactions on the anode, the cathode, or both electrodes simultaneously. For example, microorganisms could oxidize organic compounds and deliver electrons to the anode and other microorganisms present on the cathode could catalyse the reduction of hydrogen ions to hydrogen gas^12^. An external input voltage is applied to drive the reactions since the overall redox reaction is thermodynamically unfavourable. Microorganisms have different mechanisms for transferring electrons to or from electrodes. Some microorganisms have electrochemically active redox proteins on their outer membrane which can transfer electrons directly to the electrode. For example, Kim, *et al*.^13^ showed that *Shewanella purefaciens* could oxidize lactate and transfer electron to solid electrode in absence of a mediator. Reguera, *et al*.^14^ showed that *Geobacter sulfurreducens* produced conductive pili, nanowires, which could be used for transferring electrons from the cell surface to the surface of Fe(III) oxides. Rabaey, *et al*.^15^ showed that *Pseudomonas aeruginosa* produced soluble redox mediators, electron shuttles, which could be used by themselves or by other bacteria to enhance electron transfer between the cells and solid electrodes.

On a cathode, hydrogen can be produced abiotically^3^, biotically^1^, or enzymatically^16^. Microorganisms that produce hydrogen contain hydrogenases that catalyse the reversible reaction of 2 H^+^ + 2e^−^ ↔ H_2_. It was previously shown that purified hydrogenases can enhance hydrogen production on a carbon electrode^17–19^. However, the enzymes are very unstable and usually lose their catalytic activity over time. Therefore, using whole cells can help to improve the stability of the system and enhance the hydrogen production reactions. Rozendal, *et al*.^1^ was the first to investigate the possibility of producing hydrogen using the ability of microorganisms for taking up electrons from the cathode in a MEC. They showed that bioanodes enriched on acetate and hydrogen could catalyse hydrogen production when the polarity of the electrodes was reversed.

Several microorganisms in pure cultures have been shown to catalyse cathode reactions in MECs. For example, *Geobacter sulfurreducens* was shown to catalyse hydrogen production at a potential between −0.8 and −1.0 V versus Ag/AgCl^20^. *Desulfovibrio* spp. have also been shown to catalyse H_2_ production^21^ and *Methanobacterium* spp. appear capable of catalysing reduction of CO_2_ to CH_4_ with a cathode as electron donor^22^. In enrichment cultures, many microbial taxa have been found on biocathodes and it is unclear which are involved in catalysing the electrochemical reactions. For example, on H_2_-producing cathodes, Croese, *et al*.^23^ described a community consisting of 46% Proteobacteria, 25% Firmicutes, and 17% Bacteroidetes. *Desulfovibrio* spp. appeared to play a key role in this community. Batlle-Vilanova, *et al*.^24^ enriched biocathodes dominated by *Hoeflea sp*. and *Aquiflexum sp*. *Methanobacterium* spp. have been observed on CH_4_-producing biocathodes^8,25^, and *Acetobacterium* spp. and *Acetoanaerobium* spp. have been observed on acetate-producing biocathodes^25–28^. These microorganisms are known hydrogenotrophs, which suggest that H_2_ is acting as an intermediate in electrode-attached biofilms producing CH_4_ and acetate. It is unclear, however, if these or other microorganisms in the diverse cathode communities, or even free enzymes^29^, catalyse H_2_ production on the cathode.

Biocathodes are usually started-up using a mixed culture inoculum, e.g. from wastewater and anaerobic digester sludge. However, the enrichment process is slow and can take several months^25^. One strategy to facilitate start-up is to pre-enrich a suitable microbial community and use it as inoculum. Previous research suggest hydrogenotrophic microorganisms are important on biocathodes and some studies have used pre-enriched H_2_-oxidizing bioanodes^1,30^ and hydrogenotrophic methanogens^31^ with promising results. However, detailed information about the microbial community composition in the inoculum and studies on which microorganisms in the inoculum are retained on the biocathode are lacking. Many different groups of hydrogenotrophic microorganisms exist and there is a lack of knowledge about how widespread the ability to catalyse biocathode reactions is.

The first goal of this study was to screen for the ability of four hydrogenotrophic cultures, enriched using different electron acceptors, to catalyse cathode reactions. One acetate-oxidizing methanogenic culture was also tested. The other goal was to investigate current generation and change in microbial community composition when some of the enrichment cultures were used as inoculums for biocathodes in MECs.

## Results

### Enrichment cultures

#### Growth

**Figure 1:**
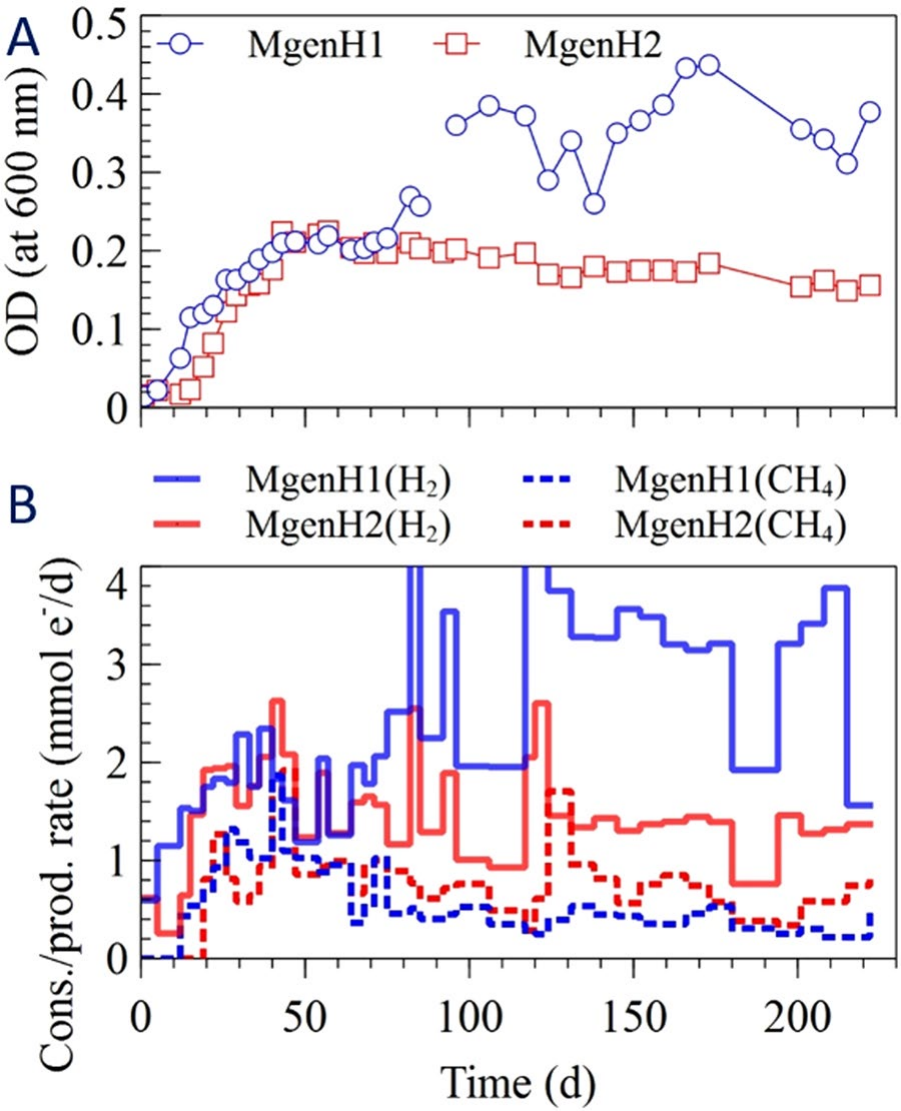
(**A**) Optical density, (**B**) H_2_ consumption rate and CH_4_ production rate in the methanogenic enrichments (MgenH).

**Figure 2:**
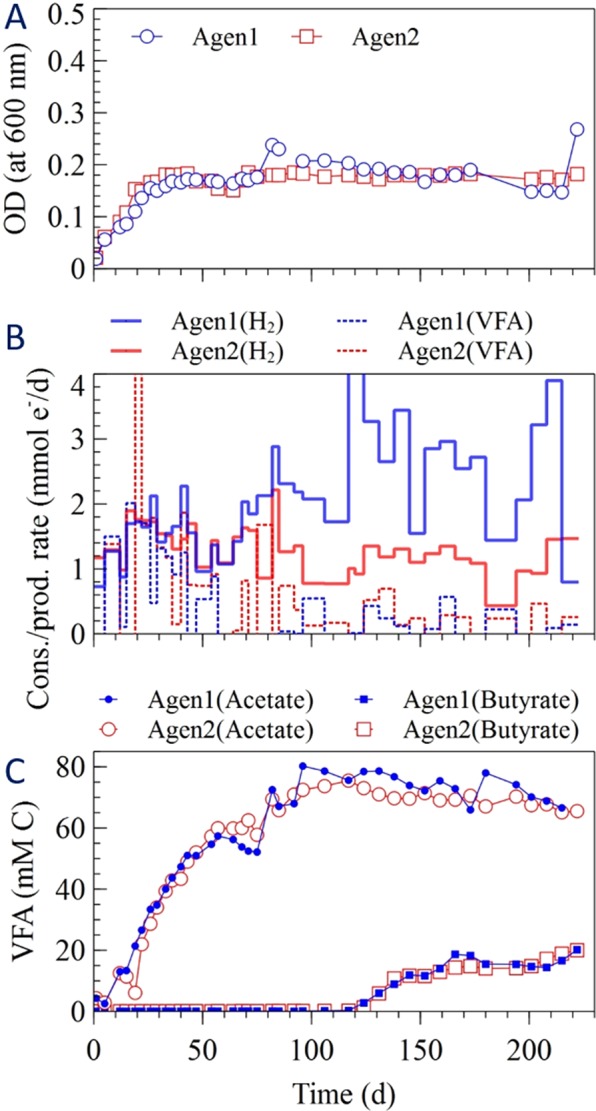
(**A**) Optical density, (**B**) volatile fatty acid (VFA) production and H_2_ consumption rates, (**C**) acetate and butyrate concentrations in the acetogenic enrichments (Agen).

**Figure 3:**
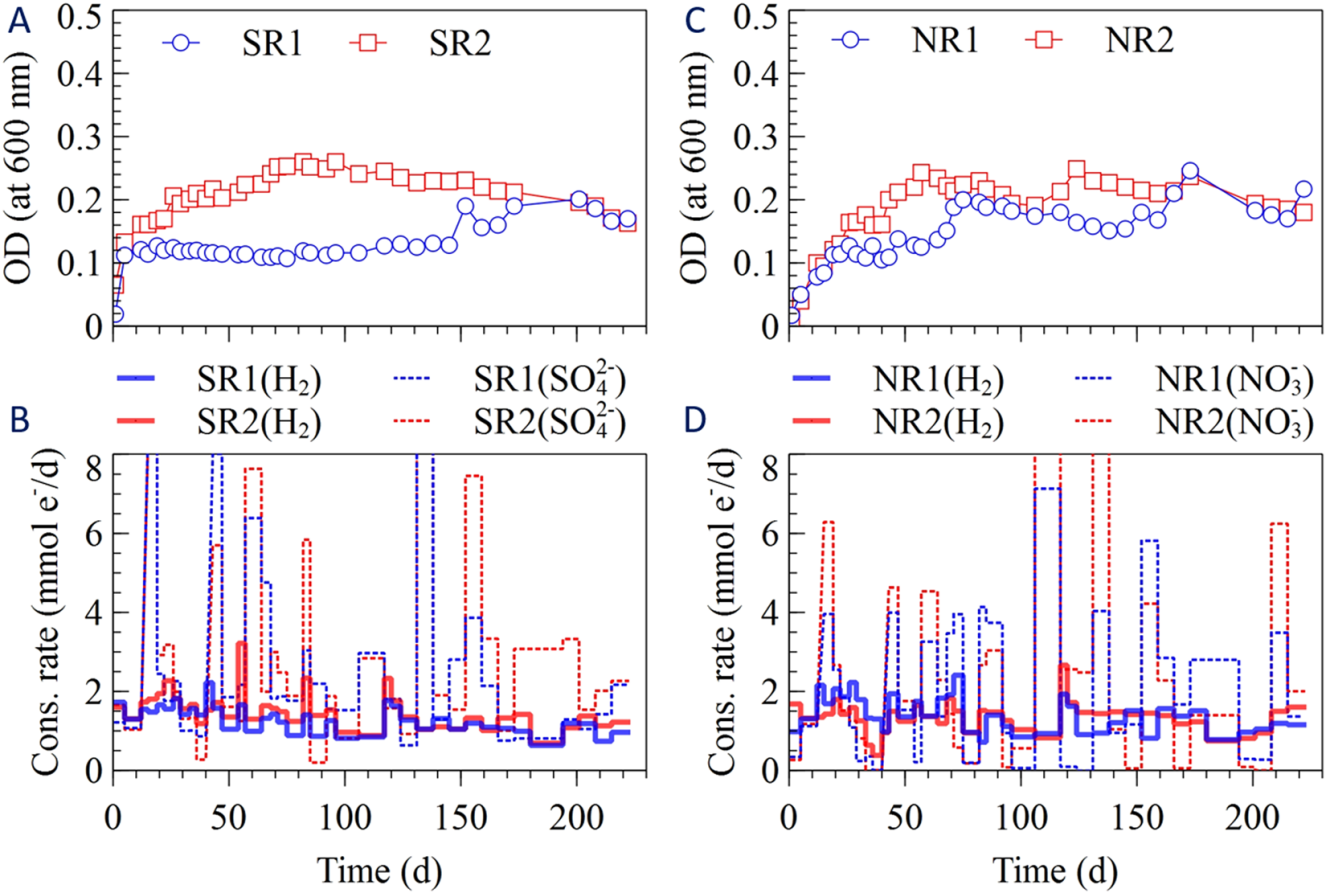
(**A**) Optical density, (**B**) SO_4_^−2^ and H_2_ consumption rates in the sulfate-reducing enrichments (SR). (**C**) Optical density, (**D**) NO_3_^−^ and H_2_ consumption rates in the denitrifying enrichments (NR).

**Figure 4:**
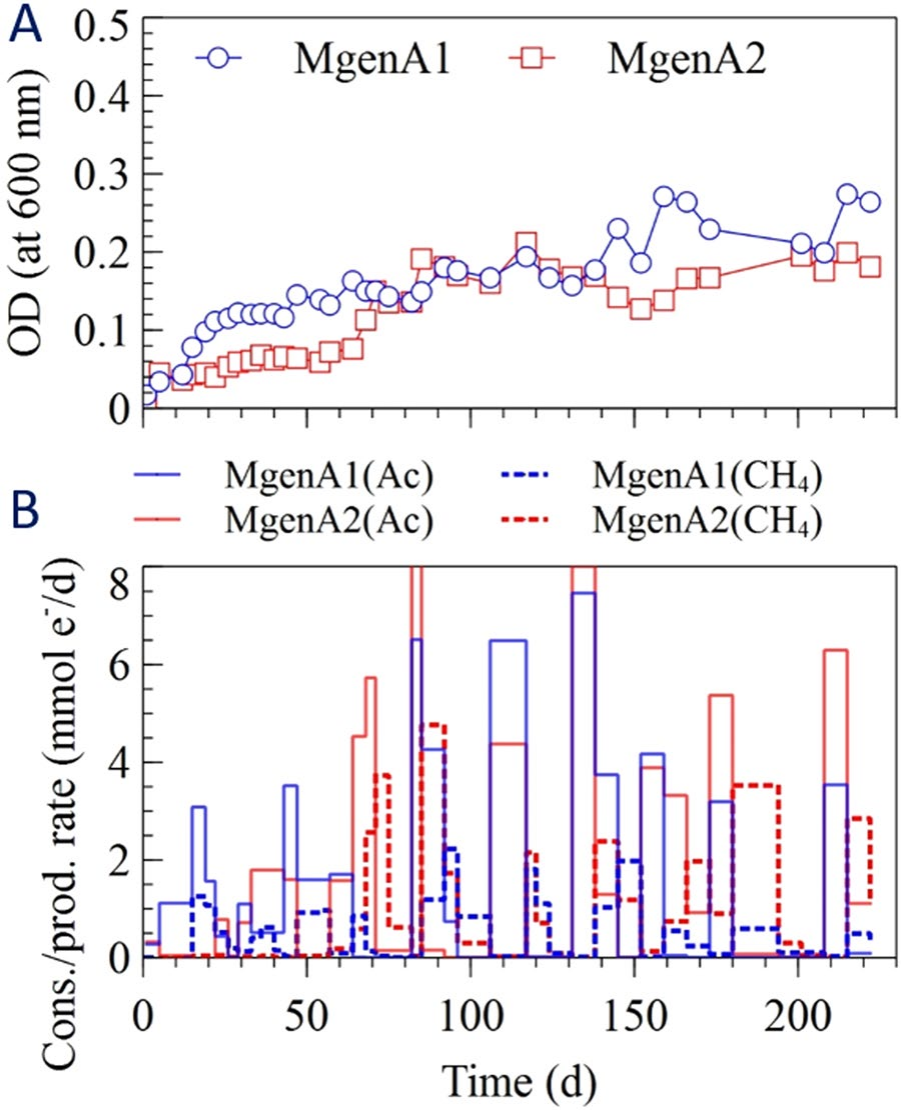
(**A**) Optical density, (**B**) acetate consumption and methane production rates in acetotrophic methanogenic enrichments (MgenA).

The change in optical density in the different enrichment cultures is shown in Figs 1–4. The cells grew quickly during the first 20 days of the experiment, except for one of the acetotrophic methanogenic enrichments (MgenA2), which had the slowest growth among the enrichments (Fig. 4). The sulfate-reducers (SR 1-2) had the fastest growth rate among the enrichments and started after 5 days (Fig. 3A). After approximately 70 days, the optical density reached to more stable values for all the enrichments suggesting that the cultures had reached a stationary phase where growth equalled decay (Figs 1–4). One of the hydrogenotrophic methanogenic enrichments (MgenH1) had an increased biomass concentration around day 90 due to removal of 140 mL for inoculation of a MEC (Fig. 1A).

#### Hydrogenotrophic methanogens (MgenH)

Hydrogen consumption rate and methane production rate are shown in Fig. 1B. Hydrogen was consumed with a rate of 0.6 mmol e^−^/d immediately after inoculation in both enrichments. The consumption rate increased slightly after 10 days for the next four weeks. Then, the rate varied between 0.8–4 mmol e^−^/d until the end of the experiment. The consumption rate was limited by the volume of H_2_ added during feeding since, in general, most of the H_2_ was consumed between two feeding occasions. In the beginning, feeding took place twice a week and in the end, once a week. MgenH1 had higher consumption rate at the end of the experiment because it contained a larger gas volume when 140 ml of liquid had been removed for inoculating an MEC. Both MgenH enrichments started to produce methane at a rate of approximately 0.8 mmol e^−^/d after 20 days. Then, the methane production rate varied between 0.2–2 mmol e^−^/d for the rest of the experiment. Very low amounts of acetate were detected in both enrichment cultures during the run.

#### Acetogens (Agen)

Figure 2 shows optical density, H_2_ consumption and volatile fatty acid (VFA) production rates, and concentrations of acetate and butyrate (the two major products) in the enrichments. VFA production rate was at its highest peak after 20 days (>2 mmol e^−^/d). Then, the production rate decreased for the next 40 days. Comparing the hydrogen consumption and VFA production shows that when hydrogen was consumed, VFA was produced. In the first 100 days of the run, acetate accumulated up to a concentration of about 70 mM-C. Then, the acetate concentration remained around 60–80 mM-C for the rest of run. Butyrate started to be produced at day 120 reaching a concentration of 20 mM-C in both enrichments at the end of the experiment.

#### Sulfate-reducers (SR)

Optical density, SO_4_^−2^ and H_2_ consumption rates in SR1 and SR2 are shown in Fig. 3A,B. In the SR enrichment cultures, sulfate was consumed at a rate of 1–1.5 mmol e^−^/d in the first 4 days after inoculation while the H_2_ consumption rate was between 1.5–2 mmol e^−^/d. Thereafter, sulfate consumption rate increased gradually over time, suggesting that the microorganisms capable of sulfate reduction increased in abundance in the enrichments. The same trend was observed for H_2_ consumption. On every occasion after addition of fresh medium to the enrichments, the sulfate consumption rate reached its highest rate. Then, SO_4_^−2^ consumption rate decreased gradually over the time as sulfate was consumed. The reason that the SO_4_^−2^ consumption rate is higher than the H_2_ consumption rate could be that the electron equivalent consumption was calculated considering only reduction of SO_4_^−2^ to H_2_S. However, in reality less reduced reaction products such as S^0^ may have been formed.

#### Nitrate-reducers (NR)

Figure 3C,D shows optical density, H_2_ and NO_3_^−^ consumption rate in the nitrate-reducing enrichments. The NR enrichments started to consume nitrate at very low rate (<0.5 mmol e^−^/d) directly after inoculation. Then, NO_3_^−^ and H_2_ consumption rates increased slightly up to 3.8–6.2 mmol e^−^/d and 1.8–2.2 mmol e^−^/d respectively at day 20 which showed that denitrifying microorganisms grew in the enrichments. The nitrate consumption rate was calculated based on the assumption that all nitrate was converted to nitrogen gas; however, nitrate and nitrous oxide could be also have been produced. Therefore, the amount of electron equivalents that were consumed is higher than the consumption rate of H_2_. Consumption of NO_3_^−^ in both NR enrichments increased significantly whenever fresh nutrient medium was supplied. Then, the rate was decreased over the time until all the NO_3_^−^ was consumed.

#### Acetotrophic methanogens (MgenA)

Optical density, acetate consumption rate, and methane production rate in the MgenA1 and MgenA2 enrichments are shown in Fig. 4. Significant increase in acetate consumption rate (1.2 mmol e^−^/d) after 5 days in MgenA1 shows that the microorganisms started to grow from the beginning of the experiment when acetate was the only electron donor available. However, in MgenA2, acetate consumption did not occur during the first 20 days (<0.3 mmol e^−^/d). This is also confirmed by the optical measurements, which showed a slow growth in MgenA2 during the first 20 days. In both enrichments, acetate consumption rate was at its peak when the new fresh medium, containing 20 mM acetate, was added. The rate gradually decreased over the time when the acetate was consumed. Methane started to be produced in MgenA1 enrichment after 15 days (1.2 mmol e^−^/d) which was faster compared to MgenA2 that started after 55 days (0.2 mmol e^−^/d). At the end of the experiment, the methane production rate in both MgenA cultures was significantly higher than the other methanogenic enrichments (MgenH 1–2). At the end of the experiment, methane production rate in MgenA2 was higher compared to MgenA1 because of 140 mL of the liquid had been removed from MgenA1 to inoculate an MEC.

#### Linear Sweep Voltammetry (LSV) and exchange current

Bioelectrochemical activity of the enrichments was evaluated using LSV. The exchange current density (i_0_) was calculated based on the Tafel equation for overpotentials greater than 0.4 V versus open circuit potential (OCP). Exchange current density for the different enrichments is showed in Figure S1. It increased after about one month in all enrichments except for NR and MgenA1 which increased after 47 and 96 days, respectively. In most of the subsequent tests, i_0_ in the presence of the microorganisms had higher value compared to the control. The improvement in exchange current density in the presence of microorganisms showed the ability of microorganisms to catalyse reduction reactions on a cathode. Linear sweep voltammograms of each enrichment at each time point results are shown in Figure S2.

### Operation of MECs

#### Current production in MECs

**Figure 5:**
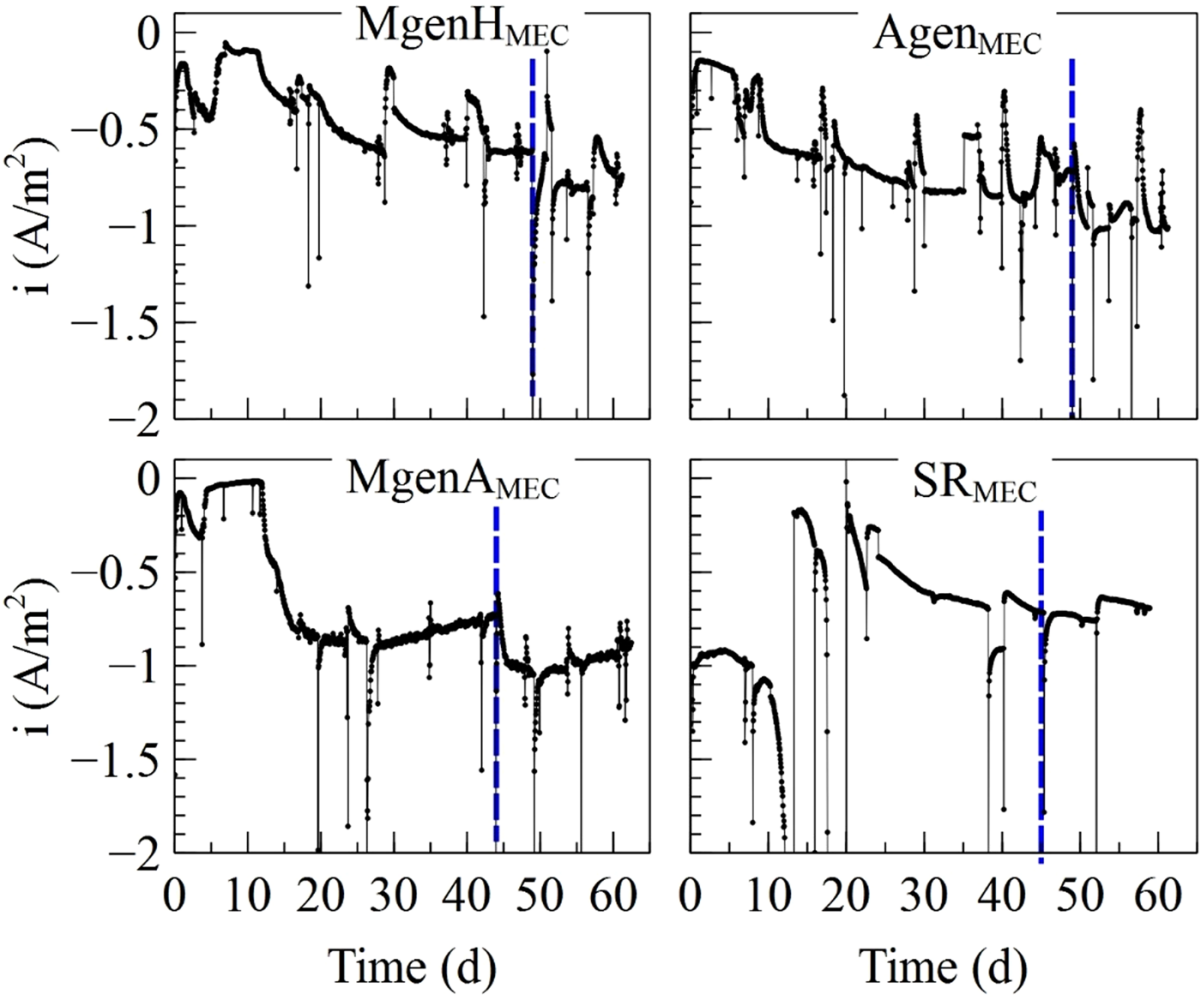
Current generation in MECs inoculated by MgenH1, Agen1, MgenA1, and SR1. The cathode potential was lowered from −0.65 V to −0.8 V versus SHE on day 49 in MgenH and Agen and on day 44 in MgenA and SR MECs as indicated by the dashed vertical lines.

Four different MECs, inoculated with microorganisms from the MgenH1, Agen1, SR1, and MgenA1 enrichments, were operated over 8 weeks in order to investigate the catalytic ability of selected enrichments on a cathode over time. Figure 5 shows the current that was generated. The MgenH_MEC_ generated a current density of about 0.2 A/m^2^ directly after inoculation followed by an increase up to 0.4 A/m^2^ during the next 6 days. However, the current dropped to 0.1 A/m^2^ for the next 5 days, followed by an increase to approximately 0.6 A/m^2^ after 29 days from the start-up. After 49 days, the potential was switched from −0.65 V to −0.8 V versus SHE and the current increased up to 0.8 A/m^2^. In Agen_MEC_, the current was generated directly after inoculation with a rate of about 0.2 A/m^2^ for the first 6 days. For the next 44 days, the current increased up to around 0.8 A/m^2^ before changing the potential from −0.65 V to −0.8 V versus SHE. After lowering the potential, the current increased up to around 1 A/m^2^. In MgenA_MEC_, current was generated at 0.1 A/m^2^ directly after inoculation followed by an increase up to 0.3 A/m^2^ after 4 days. Then, the current decreased to around 0.02 A/m^2^ for next 8 days. However, 12 days after inoculation, the current started to increase, and it reached to 0.8 A/m^2^. The current was varied between 0.8 to 0.9 A/m^2^ until the potential was switched from −0.65 V to −0.8 V versus SHE. After switching the potential, current generation increased to 1 A/m^2^ and varied between 0.8 to 1 A/m^2^ till the end of the experiment. The SR_MEC_ behaved differently to the other MECs. The current reached up to 1 A/m^2^ already in the first 7 days after the inoculation. However, it dropped to 0.2 A/m^2^ before an increase again to a more stable value at around 0.7 ± 0.05 A/m^2^ for the rest of the experiment. Decreasing the potential from −0.65 V to −0.8 V versus SHE did not have a noticeable effect on current generation in SR_MEC_ as opposed to the other MECs in this study.

Low concentrations of butyrate and lactate representing 4–5% of the charge passed in the MEC were observed in the SR_MEC_ but not in the other MECs. Concentrations of other VFAs (formate, acetate, and propionate) were negligible. Hydrogen and methane were likely produced but could not be quantified, possibly because of small leakages through electrode- and membrane connections.

#### Cyclic Voltammetry (CV)

**Figure 6:**
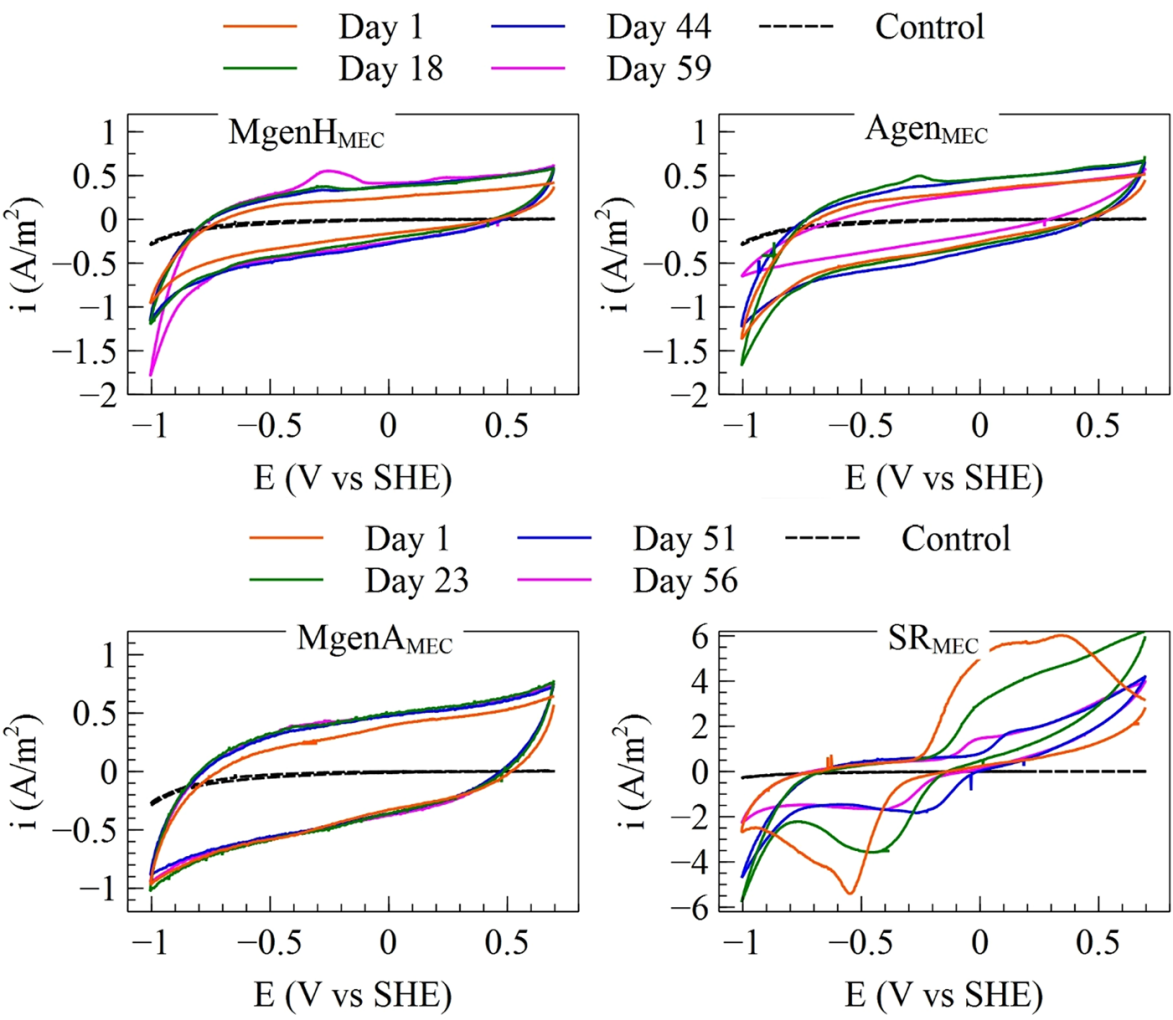
Five different CV tests carried out for MgenH-, Agen-, MgenA-, and SR-MECs. The control CV was carried out without the presence of microorganisms.

Figure 6 shows CV tests that were carried out during the operation of the MECs in order to evaluate the bioelectrochemical activity of the biocathodes. In all MECs, biological activity on the biocathode was observed directly after inoculation at day 1. In MgenH_MEC_, the hydrogen evolution peak near −1 V versus SHE gradually shifted to higher current at more positive potentials with longer operation time of the reactors. In Agen_MEC_, the current increased slightly at the potential of −1 V versus SHE after 18 days. However, at the end of the MEC operation, the current peak at −1 V versus SHE was much lower compared to previous CV tests even though the MEC was operated at −0.8 V versus SHE the last 12 days of the experiment. In SR_MEC_, a clear reduction peak was observed at −0.55 V versus SHE after inoculation. The next CV tests carried out on day 26, day 54, and day 66, showed reduction peaks at −0.42 V, −0.22 V, and −0.36 V versus SHE, respectively. The current that was generated at −1 V versus SHE at day 26 and day 54, increased noticeably and shifted more towards −0.9 V versus SHE compared to the beginning of the experiment. However, at day 66 the current that was generated at −1 V versus SHE was decreased markedly. The CV tests for MgenA_MEC_ did not show any change over the operation period even though a current of about 1 A/m^2^ was generated in the MEC at the end of the experiment. This indicates that the microbial community was electrochemically active on the cathode, however, catalysis of cathodic reactions did not improve over time.

### Microbial community analysis

**Figure 7:**
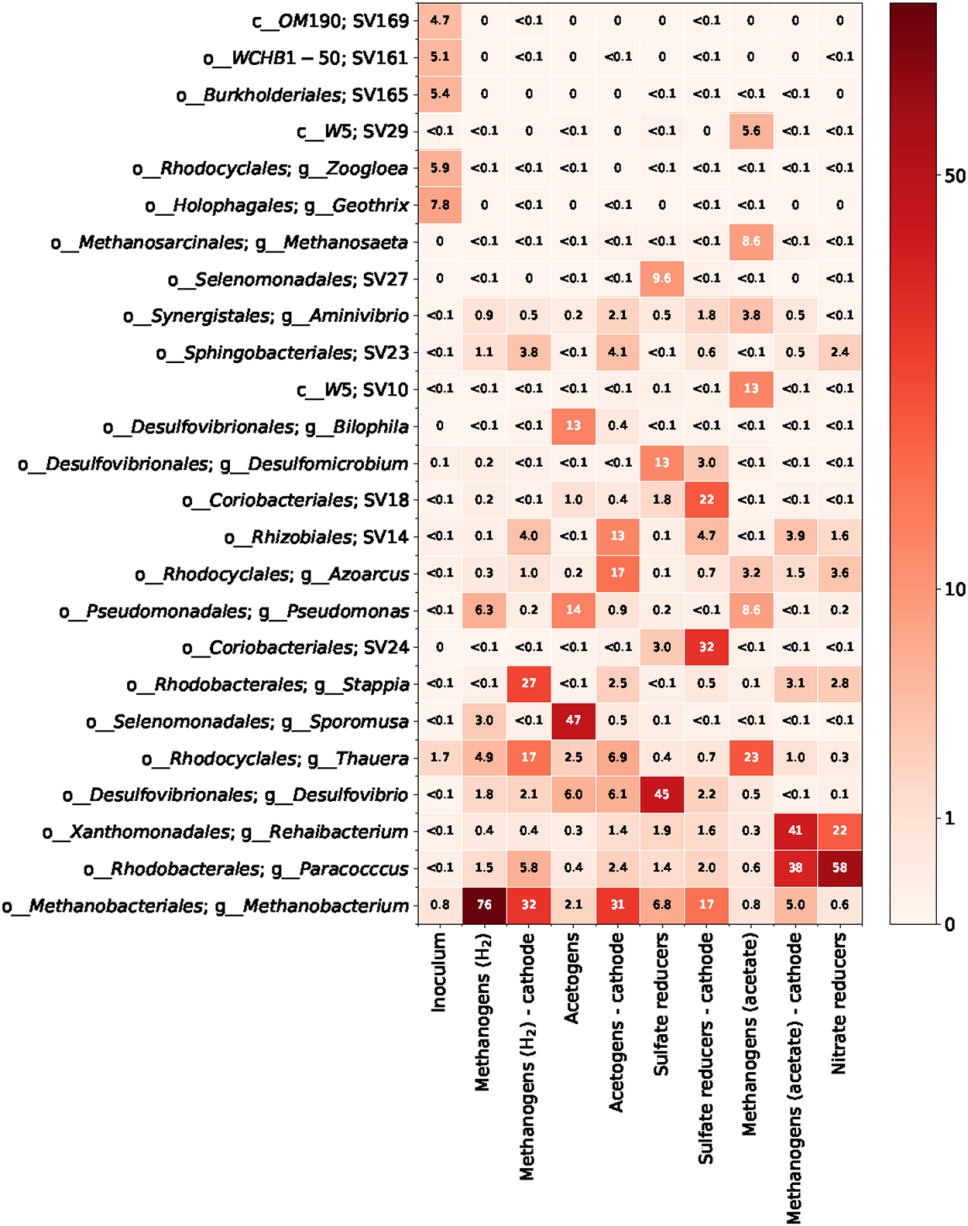
Relative abundance of the 25 most abundant taxa in the inoculum, suspended enrichments, and on the cathodes after 63 days of operation of the MECs. SV followed by a number means that the sequence could not be classified to a known genus.

A summary heatmap showing the relative abundance of the most abundant taxa in the suspended enrichment cultures and on the cathodes in the MECs is shown in Fig. 7. The values for the suspended cultures are calculated by merging the results from all samples taken during the enrichment. More detailed heatmaps for each enrichment culture are shown in Figure S3 (supplementary material). The inoculum contained a very diverse community. *Geothrix*, *Zoogloea*, and three SVs unclassified at the genus level but belonging to *Burkholderiales*, *WCBH1-90*, and *OM90* were dominating. Neither of these taxa remained detectable in the enrichments, instead other taxa increased in abundance.

The MgenH enrichments were dominated by *Methanobacterium sp*. (76%), which is known as an archaeon using H_2_ and CO_2_ to produce methane in anaerobic digesters^32^. The genera *Thauera*, *Pseudomonas*, and *Sporomusa* were also occasionally found at relative abundances over 2%. *Sporomusa* spp. are known as hydrogenotrophic acetogens and are found in different anaerobic environments^33,34^. *Thauera* spp. are often found in aerobic and denitrifying environments^35^ and can use several substrates. It may have consumed and scavenged low levels of oxygen, which may have leaked into the cultures. *Pseudomonas* may also have served as oxygen scavengers^36^.

In the Agen enrichments, the most abundant sequences were mainly affiliated to *Sporomusa* (47%)*. Bilophila*, *Pseudomonas*, and *Desulfovibrio* were also abundant. *Bilophila* is anaerobic bacteria^37^. *Desulfovibrio* spp. reduce sulfate with hydrogen or organic acids as electron donor^38^ and an anode as electron acceptor^39^.

In the SR enrichments, *Desulfovibrio* was the dominating genus (45%). *Desulfomicrobium* sp. (13%) and SV27 of the *Selenomonadales* order were also abundant, as well as *Methanobacterium*. *Desulfomicrobium* spp. are sulfate-reducing anaerobic bacterium, which can utilize hydrogen and several organic electron donors^40^. The *Selenomonadales* order contain homoacetogens such as *Sporomusa* sp.

In the MgenA enrichments, *Methanosaeta*, which is capable of producing methane from acetate^41^, was the dominating methanogen, however only present at 8.6% of relative abundance. *Thauera*, and *Pseudomonas* were also abundant and may have served as microaerobic scavengers of acetate and oxygen. Two unclassified sequences (SV10, SV29) belonging to the W5 class of the *Cloacimonetes* phylum were also abundant.

The NR enrichments were dominated by *Paracoccus* (58%) and *Rehaibacterium* (22%), which suggests they were capable of hydrogenotrophic denitrification. *Paracoccus* sp. has previously been observed on denitrifying biocathodes^42^. *Stappia*, *Azoarcus*, and SV23 of the *Sphingobacteriales* order were also abundant. *Stappia* and *Azoarcus* spp. are known to denitrify with a range of electron donors^43,44^.

The microbial communities that developed on the cathodes in the MECs were different from the suspended enrichment cultures. *Methanobacterium* sp. was an important taxon on all cathodes. The relative abundance ranged from 5% in the MgenA_MEC_ to 32% in the MgenH_MEC_. *Methanobacterium* spp. have been observed on biocathodes in MECs in several previous studies^25,45^. *Desulfovibrio* sp. were present on all biocathodes although it decreased in abundance in the MEC inoculated with SR. It was previously shown that *Desulfovibrio* sp. are capable of catalysing H_2_-production on a cathode with a columbic efficiency close to 100%^21^ and sulfate-reducing bacteria appear to play a key role in microbial electrosynthesis^46^. *Paracoccus*, *Thauera*, and *Stappia* were also present at high relative abundance on several of the cathodes. These taxa likely served as scavengers of oxygen leaking into the MECs. The unclassified SV23, belonging to the *Sphingobacteriales* order and SV14 of the *Rhizobiales* order increased in relative abundance on all cathodes. *Sphingobacteriales* spp. have previously been found on biocathodes and may be involved in catalysing hydrogen generation^26^. *Rhizobiales* spp. were observed in methane-producing microbial electrosynthesis reactors where they may have functioned as methane oxidizers^47^. Two SVs of the *Coriobacteriales* order were particularly abundant (54% in total) on the cathode in the MEC inoculated with the SR culture. Bacteria in this order have previously been shown to play a role in the catalysis of anaerobic reduction reactions on biocathodes^48^. For the MEC inoculated with Agen culture, it is worth noting that *Sporomusa* sp., which were highly abundant in the suspended enrichment culture (47%) and are known to catalyse microbial electrosynthesis of acetate^6^, made up only 0.5% of the reads on the cathode.

**Figure 8:**
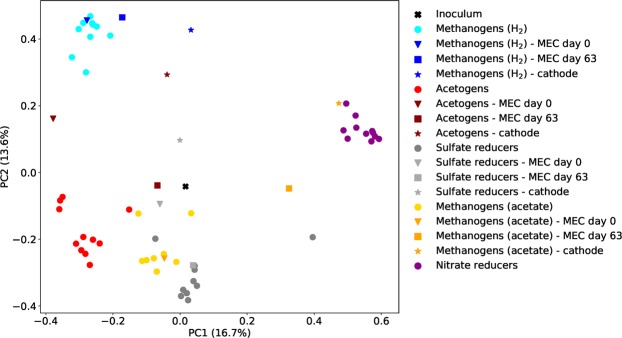
Principle coordinate analysis based on a matrix of pairwise dissimilarities between samples calculated the beta component of Hill numbers of order 1^71^.

The similarity of the microbial communities in different samples were visualized using principal coordinate analysis (Fig. 8), which showed that samples from cultures enriched on the same substrates grouped together but were clearly separated from samples enriched on other substrates. The samples collected after 63 days of MEC operation are different from the suspended enrichment and from each other. An exception is the SR enrichment, were the liquid sample from the MEC is very similar to the suspended enrichment culture while only the cathode sample has diverged. The different cathode samples do not cluster together but depend on the inoculum.

## Discussion

### Enrichments

In general, the five enrichment cultures functioned as intended. The hydrogenotrophic cultures consumed hydrogen and produced methane (MgenH) and acetate (Agen), and reduced sulfate (SR) and nitrate (NR) while the acetate-oxidizing culture (MgenA) produced methane. The enrichments also formed distinct microbial communities depending on their electron donor and electron acceptor (Fig. 8). In the Agen and SR enrichments, addition of 2-bromoethanesulfonate was generally an effective strategy for inhibiting methanogens. However, in the end of the experimental run *Methanobacterium* appeared and methane was produced in low rates (0.02 mmol/d) which suggesting 2-bromoethanesulfonate was degraded over time. Degradation has previously been observed to occur, particularly under aerobic conditions^49^.

The Agen enrichments started to produce butyrate around day 120 when acetate had accumulated to its maximum concentration (Fig. 2C). No butyrate was detected in the other enrichments, which suggests that the accumulation of acetate and inhibition of methanogens played key roles for butyrate production. A modelling study suggested that acetate reduction to butyrate with hydrogen as electron donor is unlikely due to thermodynamic constraints^50^. The reduction of acetate to ethanol after which ethanol serves as electron donor for chain elongation of acetate to butyrate could be a possible pathway^51^. However, no ethanol was observed in the enrichments. Raes, *et al*.^52^ suggested that the slow kinetics of acetate reduction to ethanol in comparison to the ethanol oxidation rate during acetate chain elongation could explain why ethanol is not observed. However, they also suggested that butyrate might be produced via acetyl-CoA reduction with hydrogen or a cathode serving as electron donor. *Sporomusa* sp., which dominated the Agen enrichments, can produce both acetate and ethanol when fed with H_2_ and CO_2_^53^. Thus, acetate and ethanol produced by the *Sporomusa* sp. may have been used to produce butyrate by other members of the community.

### Cathode catalysis

All the enrichment cultures seemed to improve catalysis of the hydrogen evolution reaction on the cathode, although the results for the LSV tests were variable. All cultures also contained taxa that have previously been observed on microbial electrodes. *Methanobacterium* was highly abundant in the MgenH enrichment. Cheng, *et al*.^8^ showed that both a mixed culture dominated by *Methanobacterium* sp. and a pure culture of *Methanobacterium palustre* could catalyse methane production on a biocathode. Several studies have also shown that *Methanobacterium* spp. are selected for on biocathodes^25,45,54^. In the Agen enrichment, a *Sporomusa* sp. was highly abundant. Nevin, *et al*.^55^ showed that several *Sporomusa* spp. could use a cathode to produce organic acids. The SR enrichment was dominated by *Desulfovibrio* spp., which are known to catalyse H_2_ generation on biocathodes^21,46^. The MgenA enrichments, contained *Pseudomonas* spp, which are known to mediate electron transfer to an anode by production of soluble redox mediators^15^. MgenA also contained *Methanosaeta*, which has been observed on biocathodes^56,57^. The NR enrichments were dominated by *Paracoccus* spp., which have been observed on denitrifying biocathodes^42^. Thus, all the enrichments contained microbial taxa that have previously been reported to be electrochemically active. A previous study also reported that hydrogenases released by cells during culturing can adsorb to electrodes and catalyse hydrogen generation^29^. In this study, hydrogenotrophic microorganisms were enriched. These microorganisms must have contained hydrogenses, which could have been released during cell lysis.

### MEC operation

The MECs generated current densities between 0.1–1 A/m^2^ at a potential of −0.65 V versus SHE directly after inoculation. This can be compared to other strategies used to start-up biocathodes. One strategy that has been investigated in several studies is the pre-enrichment of bioanodes followed by a change in potential so the electrodes operate as biocathodes. This was used successfully by Rozendal, *et al*.^1^, who enriched a hydrogen-oxidizing bioanode, then lowered the potential to −0.7 V versus SHE and obtained a biocathode generating a current density of about 1.1 A/m^2^. Enriching bioanodes on acetate and switching to cathode operation has been a less successful strategy. Saheb-Alam, *et al*.^25^ obtained a current density of 0.016 ± 0.007 A/m^2^ and it took over 170 days of operation as a biocathode before a current density of 0.6–3.6 A/m^2^ could be obtained. In comparison, direct start-up of the biocathode from sewage and anaerobic sludge only took 83 days^25^. Pisciotta, *et al*.^58^ enriched bioanodes on acetate in a sediment microbial fuel cell. After conversion to biocathodes the electrode generated 0.02 A/m^2^ at −0.7 V versus SHE and 0.002 A/m^2^ at −0.439 V versus SHE. Higher current densities have been obtained with biocathodes started up using other types of enrichment cultures. Villano, *et al*.^31^ used hydrogenotrophic methanogens and obtained 0.75 A/m^2^ at −0.75 V versus SHE. Both the strategy and the current density was similar to the MgenH enrichment tested in our study. However, the microbial community composition was not investigated by Villano, *et al*.^31^.

Clear reduction peaks in the CV curves day 1 and onward confirms that the cultures used to inoculate the MEC contained redox active components. Among the MECs, the SR_MEC_ had a large number of redox peaks which are comparable to the ones observed by Aulenta, *et al*.^21^ for biocathodes inoculated by *Desulfovibrio* sp. However, on the biocathode in SR_MEC,_ *Desulfovibrio* sp. was identified at a very low relative abundance. Instead, in the liquid phase_,_ *Desulfovibrio* sp. was the second most abundant taxa. It has been previously shown that *Desulfovibrio* sp. contains enzymes that can directly transfer electron from a cathode^59,60^. It is possible that free enzymes in the liquid could be the reason of observing significant redox curves in the SR_MEC_. However, the *Coriobacteriales* spp., which were highly enriched on the cathode could also have catalysed cathodic reactions. Members of this order are known to be anaerobic and carry out fermentation^61^ and have also been found on biocathodes^48^.

In general, the microbial communities that developed on the cathodes in the MECs were different from the MgenH, Agen, SR, and MgenA enrichments used as inoculum. However, they were also different to each other, which shows that the inoculum had a strong effect on how the biocathodes developed during the 63 days of operation. One similarity was the increase in *Methanobacterium* sp. on all the biocathodes except MgenH_MEC_, where it was already present in high relative abundance in the inoculum. This further confirms previous observations that this taxon is highly selected for on biocathodes reducing CO_2_/HCO_3_^−^ ^8,25,54^.

## Materials and Methods

### Inoculum, nutrient medium, and enrichment setup

Duplicate glass bottles (325 mL total volume each) were used for enriching five different cultures. Activated sludge from a municipal wastewater treatment plant (1 mL) was added to the bottles as inoculum. The bottles were filled up to 250 mL with a nutrient medium as described by Marshall, *et al*.^26^. The goal was to enrich hydrogenotrophic cultures performing methanogenesis, acetogenesis, sulfate reduction, nitrate reduction, as well as an acetate-oxidizing methanogenic culture. To accomplish this, the nutrient medium was amended with different electron acceptors and in some cases 2-bromoethanesulfonate to inhibit methanogens, as described in Table S1. The bottles were sealed with rubber caps and the head space (70 mL) was sparged with Ar/CO_2_ gas (85%/15%) to remove oxygen. Then, the head space of the hydrogenotrophic bottles were filled with pure hydrogen gas at an overpressure of 160–180 kPa. During sampling, the amount of liquid that was extracted from the enrichment cultures was replaced by fresh medium. Every 3 weeks, fresh medium (20 mL) containing 20 mM NaSO_4_, NaNO_3_, and sodium acetate was added to the sulfate-reducing, denitrifiers and acetate-oxidizing enrichments. Sterile syringes (12 mL) and needles were used for collecting samples, sparging of gas, and addition of fresh medium for each enrichment.

### Analytical methods

The gas phase was analysed by gas chromatography (micro-GC, Agilent) one/two times a week. Samples from the liquid were collected one/two times a week and were analysed by high performance liquid chromatography (HPLC) equipped with a UV detector (Shimadzu) and an Aminex HPX-87H column (BioRad), with 5 mM H_2_SO_4_ eluent pumping at 0.5 mL/min. Cell growth was measured using optical density (OD) measured at 600 nm wavelength which correlates directly with the cell concentration^62,63^.

### Electrochemical screening

Linear sweep voltammetry (LSV) was conducted occasionally with 4 mL mixed liquor collected from the enrichment cultures. The test was carried out in a double-chamber electrochemical cell (each chamber being 2 mL) containing a cation exchange membrane (CMI-7000, Membranes International Inc.), graphite foil electrodes, and a Ag/AgCl reference electrode. LSV was swept from open circuit potential (OCP) to −1.2 V versus SHE at a scan rate of 2 mV/s. Exchange current density was calculated using the Tafel equation $({\rm{\eta }}={\rm{A}}\times \,\mathrm{ln}(\frac{i}{{i}_{0}}))$ based on the region of the voltammogram representing an overpotential greater than 0.4 V versus OCP. The symbols in the equation have the following meanings: ${\rm{\eta }}$ is the overpotential, A is the Tafel slope, $i$ is the current density, and ${i}$_0_ is the exchange current density.

### MEC setup

A glass double-chamber MEC, with a total volume of 340 mL in each chamber, was used to test the ability of some of the enrichment cultures to colonize a cathode. The cathode chamber was filled up to 280 mL with a mixed liquid from the enrichment bottle (140 mL) and the nutrient medium buffer (140 mL) described above. Bicarbonate or hydrogen ions were the only electron acceptor available in the cathode chamber. A graphite foil electrode (Alpha Aesar, 43083-1 mm thick, 39.2 cm^2^) was installed as working- and counter electrode in each chamber. An Ag/AgCl reference electrode with an offset of 0.197 V versus the standard hydrogen electrode (SHE) was installed in the working-chamber. The two chambers were separated by a cation exchange membrane (CMI-7000, Membranes International Inc.). The working electrode potential was controlled using Wenking M lab potentiostat and the current was recorded by MlabSci470c sequencer multichannel potentiostat software (version 4.7.0). Since the electrode potential has a strong effect on the magnitude of the cathodic current^31,64^, the MEC were operated at both −0.65 V and −0.8 V versus SHE. The bioelectrochemical activity of the cathode was investigated using cyclic voltammetry (CV). CV was done with scan limits of 0.7 V and −1.0 V versus SHE at a scan rate of 1 mV/s.

### Microbial community analysis

Samples (10 mL) from the enrichment cultures were collected on a sterile membrane filter (Sartorius Stedim Biotech, 47 mm ϕ, 0.2 μm) for microbial community analysis. Moreover, biocathodes were harvested from MECs for microbial community analysis at the end of the experiment. Samples were stored at −20  °C prior to DNA extraction. DNA was extracted using the Fast DNA kit for soil (MP Biomedical). The 16S rRNA genes were amplified in duplicates using the forward primer 515’F (GTGBCAGCMGCCGCGGTAA) and the reverse primer 806 R (GGACTACHVGGGTWTCTAAT) to amplify V4 region sequences of the bacterial and archaeal 16S rRNA genes^65^ with dual index labelling according to Kozich, *et al*.^66^. Duplicate PCR reactions were carried out in 20 μL volume using 1 μL of target DNA, 17 μL of AccuPrime Pfx SuperMix (Life Technologies), and 1 μL of the forward and reverse primers, respectively. The PCR was conducted using a Bio-Rad T100 Thermal Cycler with a program consisting of activation (95 °C, 5 min); 30 cycles of denaturation (95 °C, 20 sec), annealing (50 °C, 20 sec) and elongation (68 °C, 60 sec), and final elongation (68 °C, 10 min). The products were purified (MagJET NGS Cleanup and Size Selection Kit, ThermoFischer Scientific), normalized per concentration, as measured by a Qubit Fluorometer (ThermoFischer Scientific), and pooled prior to sequencing on an Illumina MiSeq using the Miseq Reagent Kit V3.

The sequence reads were processed in USearch (v. 10) using the Unoise algorithm^67^ to generate sequences variants (SVs), which are analogous to operational taxonomic units^68^. Taxonomic classification was done using the Sintax algorithm^69^ with the Midas database^70^ (v. 123). Raw data files were deposited to the National Center of Biotechnology Information’s (NCBI) Sequence Read Archive (SRA) database available online with study accession of PRJNA482287. Analysis of the sequencing results was carried out using qDiv (github.com/omvatten/qDiv).

## Supplementary information

Supplementary material
