# Supplementary material for: A variety of hydrogenotrophic enrichment cultures catalyse cathodic reactions
Source: Sci Rep. 2019 Feb 20;9:2356. doi: 10.1038/s41598-018-38006-3 (PMC6382808; doi:10.1038/s41598-018-38006-3)
Supplement: Supplementary file 1 — Supplementary material [file 41598_2018_38006_MOESM1_ESM.pdf]

Supplementary material

A variety of hydrogenotrophic enrichment cultures catalyse cathodic reactions

Soroush Saheb-Alam[1*], Frank Persson[1], Britt-Marie Wilén[1], Malte Hermansson[2], Oskar Modin[1]
[1]Chalmers University of Technology, Department of Architecture and Civil Engineering, Division of Water Environment Technology, SE-412 96, Gothenburg, Sweden,
[2]University of Gothenburg, Chemistry and Molecular Biology, SE-40530, Gothenburg, Sweden
Corresponding author: Soroush Saheb-Alam, Email: soroush.sahebalam@chalmers.se, Tel: +46-317722133
Correspondence: +46 31 7722133, E-mail address: soroush.sahebalam@chalmers.se

Supporting Information: 6 pages, 1 table, 3 figures

Supplementary material

Table S1 Medium compositions that were used to cultivate different type of microorganisms in different bottles. *The medium in the bottles marked with an asterisk (*) also contained 10 mM 2-bromoethanesulfonate to inhibit methanogens

**Enrichment culture**	**Electron donor**	**Electron acceptor**
Hydrogenotrophic methanogens (MgenH)	H_2	CO_2/HCO_3^-
Hydrogenotrophic acetogens (Agen)*	H_2	CO_2/HCO_3^-
Hydrogenotrophic sulfate-reducers (SR)*	H_2	20 mM $NaSO_4$
Hydrogenotrophic nitrate-reducers (NR)	H_2	20 mM $NaNO_3$
Acetotrophic methanogens (MgenA)	Acetate	CO_2/HCO_3^-

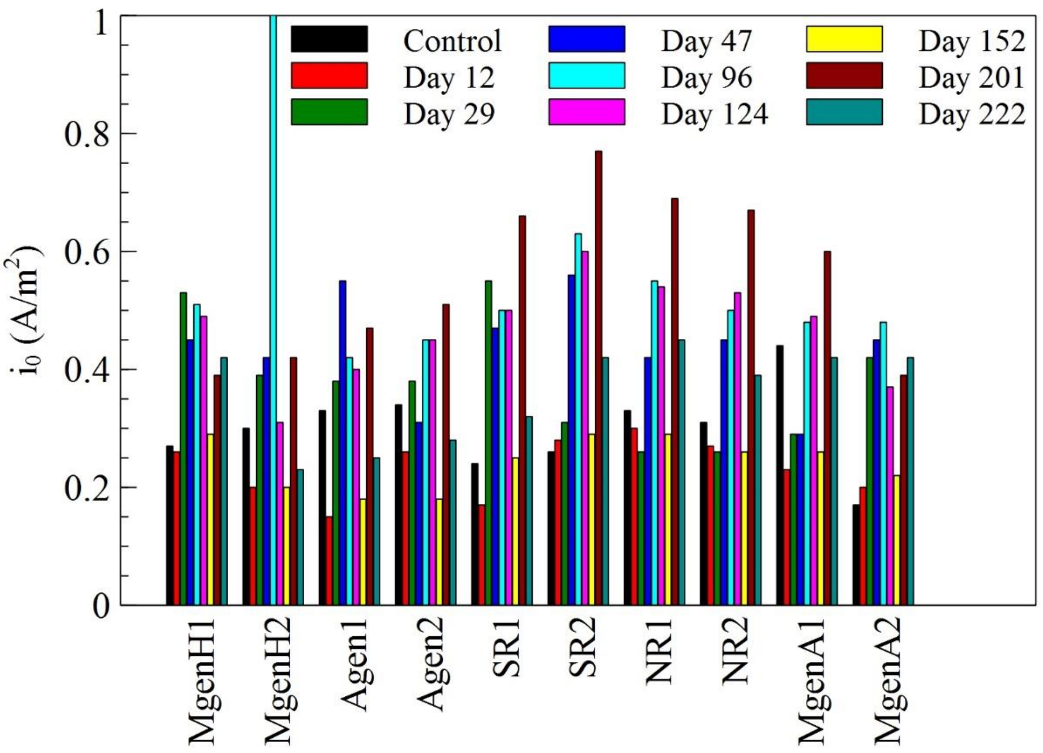

Figure S1 Exchange current density calculated based on Tafel equation for overpotentials greater than 0.4 V vs OCP for 8 LSV tests carried out during the experiment

Supplementary material

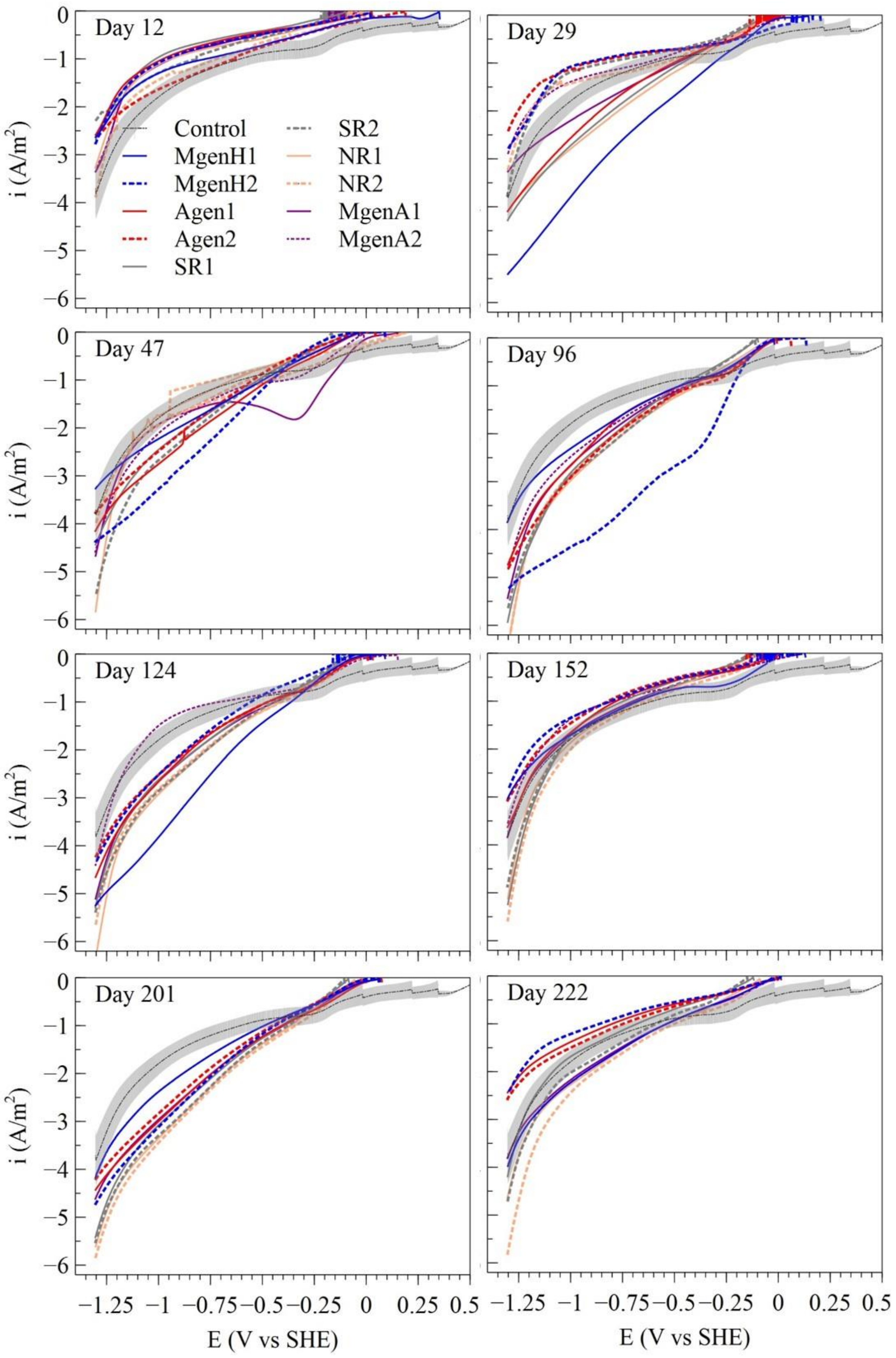

Figure S2 Linear sweep voltammetry of samples taken from enrichment culture.

Supplementary material

(MgenH)

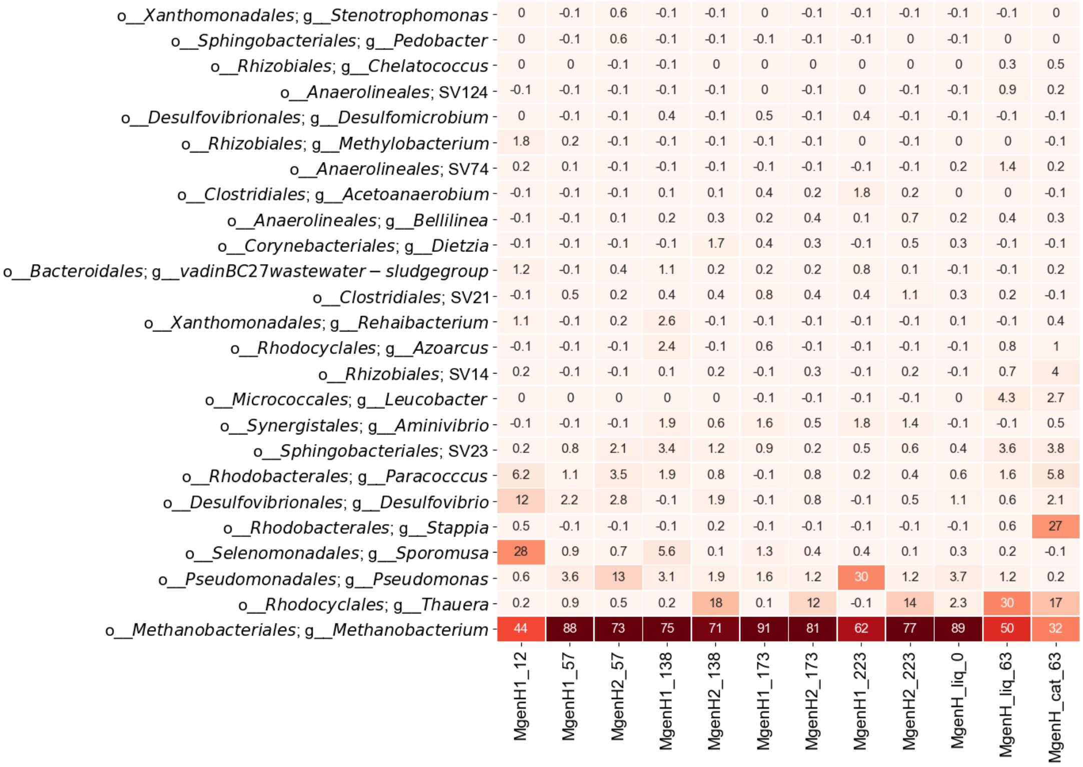

(Agen)

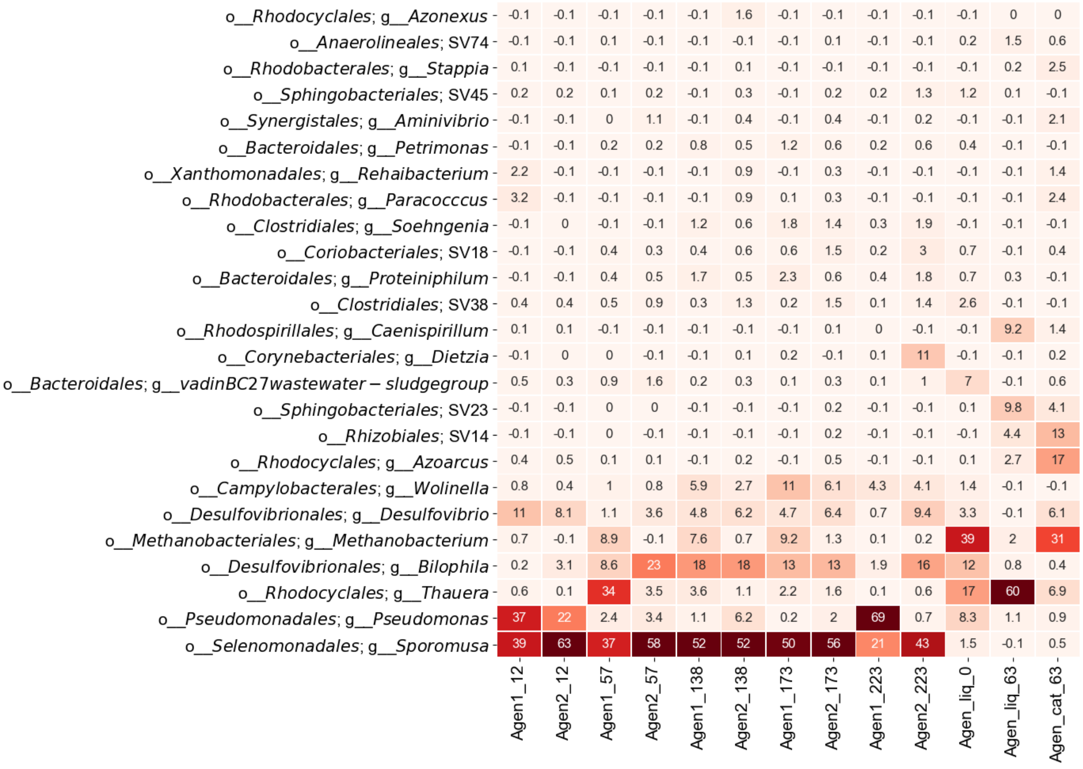

Supplementary material

(SR)

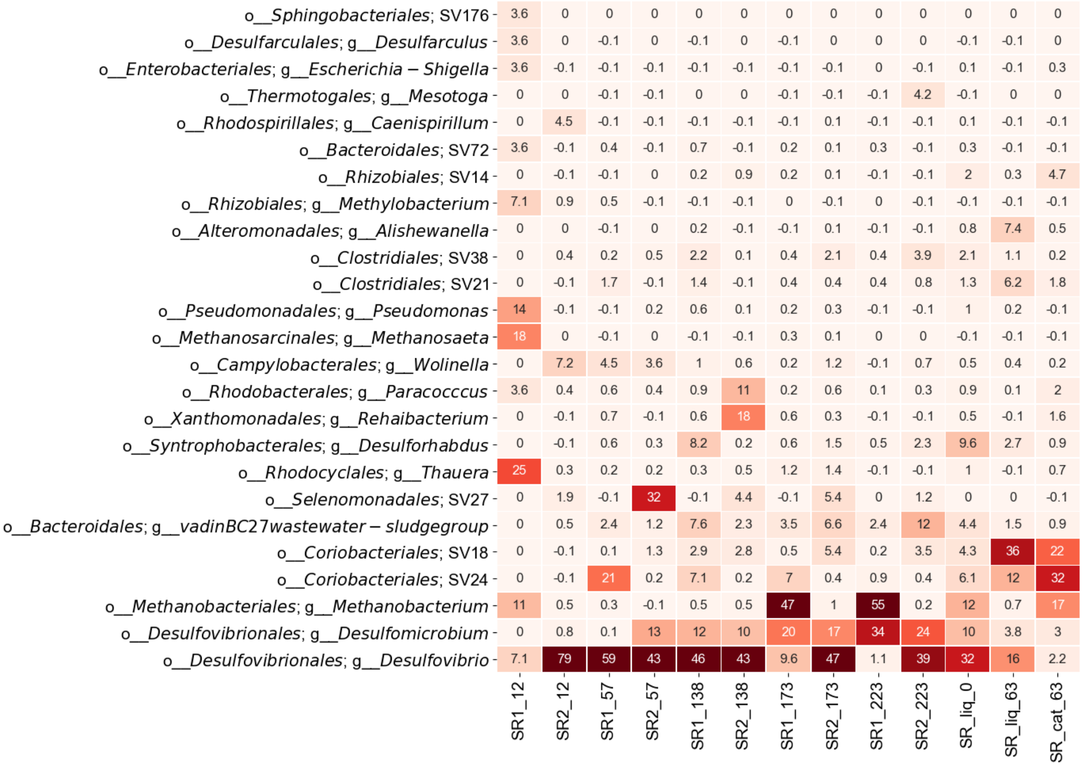

(MgenA)

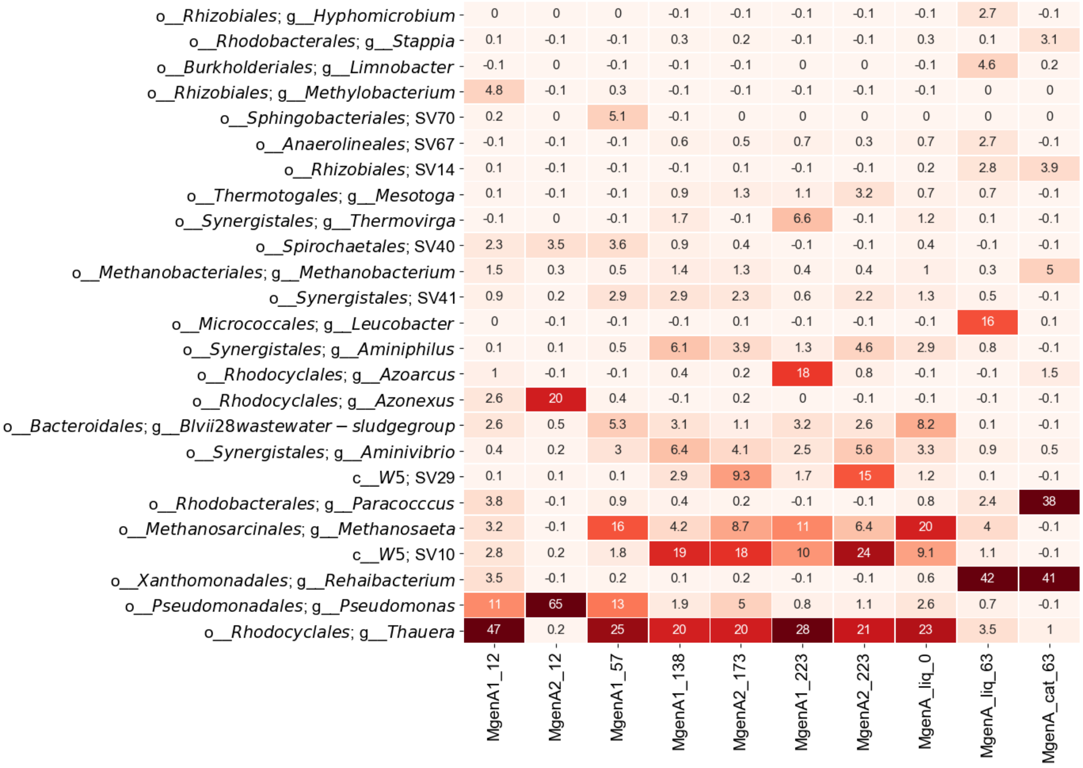

Supplementary material

(NR)

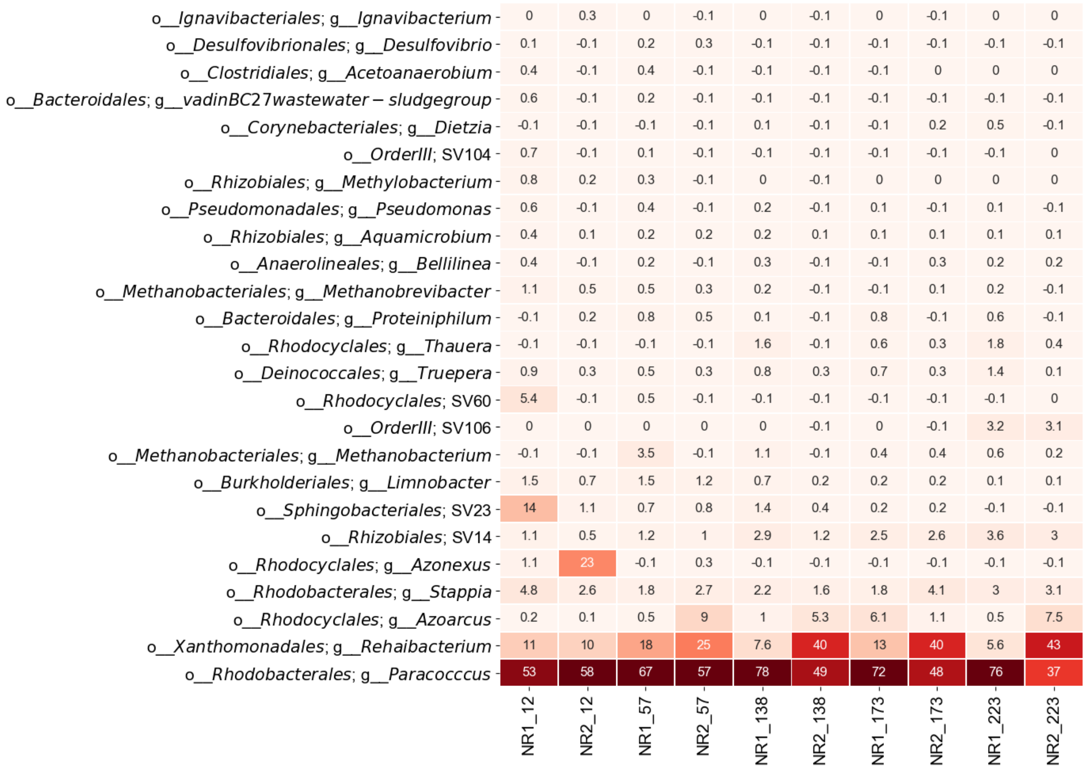

Figure S3 Detailed heatmaps of most abundant taxa in each enrichment culture and accompanying MEC.
